# Call for Convention of Medical Colleges

**Published:** 1879-03

**Authors:** 


CALL FOR A CONVENTION of all American Medical Colleges, to be held in the city of Atlanta, Ga., beginning at 10 A. M., Friday, May 2d, 1879. In general terms, the *object* of the convention is to adopt some "some uniform system of instruction more in harmony with the requirements of the age." Among the questions for discussion and decision may be mentioned, "Shall all the colleges require attendance upon three regular courses of lectures during three separate years ere admitting students to become candidates for the degree of M. D.?" Is any uniform system possible in this or other things? If so, to what extent is it possible or even desirable at the present time? Each doctor in the land doubtless has in mind an ideal medical college system. But this convention cannot act upon idealities; it can only act upon that which is practicable to all honest efficient medical schools.

